# Pathological Evidence From an Experimental Rat Model Demonstrates That Aortic Hypoperfusion Contributes to the Development of Medial Arterial Calcification

**DOI:** 10.1111/pin.70077

**Published:** 2025-12-30

**Authors:** Tomoko Sumi, Mayo Higashihara, Takuma Takeda, Taichi Imai, Yuna Tamura, Tatsuya Moriyama, Nobuhiro Zaima

**Affiliations:** ^1^ Graduate School of Agriculture Kindai University Nara Nara Japan; ^2^ Agricultural Technology and Innovation Research Institute Kindai University Nara Nara Japan

**Keywords:** animal models, aortic hypoperfusion, medial arterial calcification, vascular degeneration

## Abstract

Medial arterial calcification, ectopic deposition of calcium phosphate crystals in the media, causes aortic stiffness which is associated with the mortality of cardiovascular diseases. Previous studies clarified several factors which are related to disease progression processes, on the contrary, inducing factors of medial arterial calcification remain obscure. In this study, we performed pathological analyses of the aorta in an experimental animal model under the condition of hypoperfusion to understand unexplored events underlying medial arterial calcification. The area of calcium deposition varied with the severity of hypoperfusion, and the extent of calcium deposition was highest under conditions of severe hypoperfusion. Thinning of the media, destruction of elastic fibers, and increased transformation marker of vascular smooth muscle cells into osteoblast‐like cells were observed earlier than calcium deposition. Time‐dependent observations of the hypoperfusion‐induced aorta show the flattening of elastic fibers and death of medial cells prior to calcium phosphate deposition, followed by the formation of microvoids which were used as scaffolds for calcium phosphate crystal formation. These data showed that aortic wall hypoperfusion can be an initiating factor of calcium phosphate deposition in the arterial media.

## Graphical abstract

Time‐dependent observations of the hypoperfusion‐induced aorta show the flattening of elastic fibers and death of medial cells prior to calcium phosphate deposition, followed by the formation of microvoids which were used as scaffolds for calcium phosphate crystal formation. These data showed that aortic wall hypoperfusion can be one of the initiating factors of calcium phosphate deposition in the arterial media.

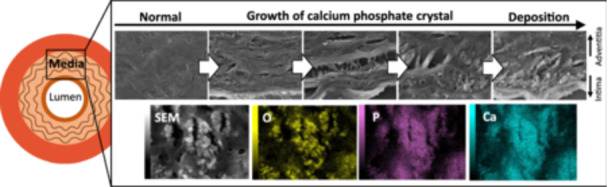

## 1 Introduction

Medial arterial calcification is a systemic chronic vascular disease in which hydroxyapatite crystals, a type of calcium phosphate, are deposited in the medial layer of arteries, independent of atherosclerosis [1, 2, 3]. Ectopic calcification in the media causes stiffness of the arterial wall which is closely associated with the mortality of cardiovascular disease. The progression of the ectopic calcification is irreversible and the estimated risk factors of the medial arterial calcification include aging, diabetes, and chronic kidney disease. Patients who have medial arterial calcification are reported to be −0.5% of adults (male/female ratio 3:2), 17%–42% of type 2 diabetes, and 27%–40% of patients with advanced chronic kidney disease [4]. At present, no pharmacological treatment has been established.

The development of medial arterial calcification is reportedly associated with cell‐independent and/or cell‐dependent processes [5, 6, 7]. In the cell‐independent process, calcium phosphate crystals are formed cell‐independently due to the gradual growth of calcium phosphate nuclei attached to elastin [7, 8]. In the cell‐dependent process, transformation of vascular smooth muscle cells (VSMCs) into osteoblast‐like cells plays a central role in the formation of calcium phosphate crystals [9, 10]. It has been reported that inflammation, extracellular matrix degeneration, and apoptosis of VSMCs are observed simultaneously with the progression of medial arterial calcification, and that the complex action of the pathological factors leads to the formation of medial arterial calcification [10, 11]. However, the inducible factors that trigger the complex progression which lead to the loss of integrity of the aorta remain unclear.

The maintenance of integrity of the aortic structure is sustained by the normal activity of aortic cells, including VSMCs. Oxygen and nutrients, which are the basic building blocks to produce energy for biological activity, are essential elements for the normal activities of aortic cells to sustain a healthy structure of the aorta. It has been reported that hypoperfusion in human aortic wall occurs due to intraluminal thrombosis and stenosis of vasa vasorum, resulting in pathological conditions in which oxygen and nutrients are not sufficiently supplied to the aortic wall [12, 13, 14]. The reproduction of the aortic wall hypoperfusion in experimental animals induce several abnormal aortic conditions including inflammation, extracellular matrix degeneration, aortic aneurysm formation, and aortic sclerosis [13, 14, 15, 16]. The pathological changes and mechanical degeneration in the aorta induced by aortic hypoperfusion are not inconsistent with those observed in human medial arterial calcification, suggesting that aortic hypoperfusion can contribute to the development of medial arterial calcification. However, the relationship between the aortic hypoperfusion and medial arterial calcification has not been fully elucidated. In this study, we performed pathological analysis of the aorta under the condition of hypoperfusion to understand unexplored events between aortic hypoperfusion and medial arterial calcification.

## 2 Materials and Methods

### 2.1 Animals

All animal experiments were approved by the Kindai University Animal Care and Use Committee and were performed according to the Kindai University Animal Experimentation Regulations (approval number: KAAG‐2022‐008, KAAG‐2025‐012). Six‐week‐old male Sprague–Dawley rats (Japan SLC Co. Ltd., Shizuoka, Japan) were maintained at a controlled room temperature of 25 ± 1°C under a 12‐h light/dark cycle. The rats in each group were fed. Food and water were available ad libitum. The diet consisted of a commercial diet (MF; moisture: 8.1%, crude protein: 23.2%, crude fat: 4.9%, crude ash: 5.9%, crude fiber: 3.3%, soluble non‐nitrogenous matter: 55.3%, Oriental Yeast Co., Ltd., Tokyo, Japan) and tap water. Forty rats were divided into five groups according to the time of sacrifice after treatment: untreated group (*n* = 8), dissected 6 h after treatment group (*n* = 8), dissected 12 h after treatment group (*n* = 8), dissected 24 h after treatment group (*n* = 8), and dissected 48 h after treatment group (*n* = 8). After a 1‐week acclimation period, the abdominal aorta was subjected to a procedure to induce aortic wall hypoperfusion and was subsequently harvested in a time‐dependent manner. All procedures were performed under anesthesia with medetomidine, midazolam, and butorphanol, and all efforts were made to minimize pain.

### 2.2 Induction of Abdominal Aortic Wall Hypoperfusion

Induction of abdominal aortic wall hypoperfusion was performed as described in a previous study [17]. The aorta below the renal vein was dissected free of adipose tissue, and the branches from the aorta were ligated with 5‐0 silk sutures (Akiyama MEDICAL MFG. Co., Ltd., Tokyo, Japan). The aortic wall was made a small incision, and an 18 G polyurethane catheter (Medikit Co., Ltd., Tokyo, Japan), shortened to a length of 9 mm, was inserted. The incision was sutured with 6‐0 monofilament sutures (Alfresa Pharma Corp., Osaka, Japan). The aortic wall above the inserted catheter was ligated in two places using 5‐0 silk sutures. The 5‐0 silk suture was left in place until the end of experiment.

### 2.3 Sample Fixation and Sectioning for Pathological Analysis

**FIGURE 1:**
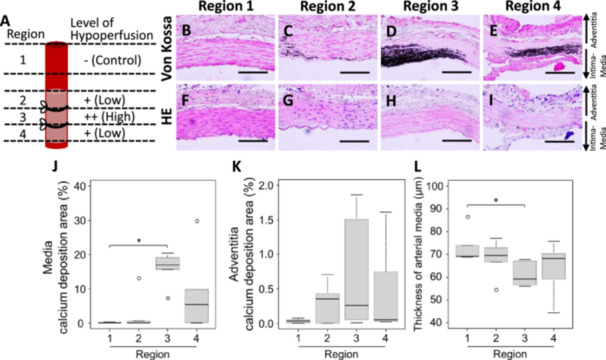
Comparison of regional calcium deposition following induction of arterial wall hypoperfusion. (A) Arterial region and level of hypoperfusion following treatment to induce arterial wall hypoperfusion. (B–E) Representative images of Von Kossa staining (scale bar = 100 μm). (F–I) Representative images of HE staining (scale bar = 100 μm). (J) Calcium deposition area in the media. (K) Calcium deposition area in the adventitia. (L) Thickness of the arterial media. Values are shown as mean ± SD, median, and interquartile range (*n* = 5). *p < 0.05, statistically significant.

The abdominal aorta was divided into four regions (Figure 1A) and fixed in 4% paraformaldehyde phosphate buffer (FUJIFILM Wako Pure Chemical Corp., Osaka, Japan). The samples were then dehydrated and embedded in tissue‐embedding paraffin wax (Sakura Finetek Japan Co., Ltd., Tokyo, Japan). Tissue blocks were sectioned at 6 µm using a rotary microtome (PR‐50; Yamato Kohki Industrial Co., Ltd., Saitama, Japan), and the sections were mounted on glass slides. The tissue sections were deparaffinized before staining and used for various pathological staining.

### 2.4 Pathological Analysis

Tissue sections were stained with Von Kossa staining, hematoxylin and eosin (HE) staining, Elastica van Gieson (EVG) staining using Sirius Red, and immunofluorescence staining. Von Kossa staining was performed using a commercial kit (CVK‐1; ScyTek Laboratories, Inc., Logan, UT, USA). Tissue sections stained by immunofluorescence were observed and photographed using an all‐in‐one fluorescence microscope (BZ‐X810; KEYENCE Corp., Osaka, Japan). Von Kossa, HE, and EVG‐stained tissue sections were observed and photographed using a microscope (CX21 LED, Olympus Corporation, Tokyo, Japan) equipped with a digital camera. Picrosirius red (PSR) stained tissue sections were observed and photographed using a polarizing filter in addition to a microscope equipped with a digital camera. Quantitative analysis was performed using ImageJ software (National Institutes of Health, Bethesda, MD, USA). Images were binarized to black and white, and the area percentage of Von Kossa staining was calculated. The area percentage of PSR staining was calculated by binarizing the yellow‐to‐red region. The destruction rate of the wavy structure of elastic fibers by EVG staining was calculated by dividing the destruction area (indicated by flattening and fragmentation of elastic fibers) by the total area of elastic fibers.

### 2.5 Immunofluorescence Staining

Tissue sections were washed with phosphate‐buffered saline with Tween 20 (PBST) and incubated with 0.1% trypsin in PBS for 10 min at 37°C for antigen retrieval. After washing, tissue sections were incubated with Blocking One Histo (Nacalai Tesque, Inc., Kyoto, Japan) for 30 min to block nonspecific binding. Then, tissue sections were incubated with primary antibodies overnight at 4°C. The next day, tissue sections were washed with PBST and incubated with fluorescently labeled secondary antibodies for 1 h in the dark. Tissue sections were washed again with PBST and then nuclear stained with 4′,6‐diamidino‐2‐phenylindole (DAPI) (Sera Care Life Sciences Inc., Milford, MA, USA). Then, tissue sections were washed with PBS and mounted with water‐soluble mounting medium. The positive areas of the stained tissue sections were calculated using the same software as used for pathological analysis, by binarizing the images into black and white and calculating the area percentage. To compare living cells, the positive area for each marker was normalized to the DAPI‐positive area. Statistical analyses were performed using values obtained from three or more regions per sample.

The antibodies used for staining are listed below. Primary antibodies: mouse anti‐non‐muscle myosin heavy chain (SMemb) (7602; dilution 1:50; Yamasa Corp., Chiba, Japan), rabbit anti‐bone morphogenetic protein (BMP)‐2 (bs‐1012R; dilution 1:50; Bioss Inc., Woburn, MA, USA), rabbit anti‐runt‐related transcription factor (Runx) 2 (M00442; dilution 1:50; Boster Biological Technology Ltd., Pleasanton, CA, USA), rabbit anti‐osteocalcin (23418‐1‐AP; dilution 1:10; Proteintech Group, Inc., Rosemont, IL, USA), rabbit anti‐osteopontin (bs‐0026R; dilution 1:50; Bioss Inc., Woburn, MA, USA), and rabbit anti‐cathepsin K (11239‐1‐AP; dilution 1:100; Proteintech Group Inc., Rosemont, IL, USA). Secondary antibodies: Goat Anti‐Mouse IgG H&L (Alexa Fluor 488) (ab150113; dilution 1:500; Abcam plc., Cambridge, UK), and Goat Anti‐Rabbit IgG H&L (Alexa Fluor 555) (ab150078; dilution 1:500; Abcam plc., Cambridge, UK).

### 2.6 Scanning Electron Microscopy (SEM) and Energy Dispersive X‐Ray Spectroscopy (EDS)

Electron microscopy and elemental analysis of the aortic wall were performed using a scanning electron microscope (SEM) equipped with energy dispersive X‐ray spectroscopy (EDS) (JCM‐7000 Neoscope; JEOL Ltd., Tokyo, Japan). Deparaffinized tissue sections were used for electron microscopy. The microscope was operated under high vacuum at an accelerating voltage of 15.0 kV.

### 2.7 Statistical Analysis

Values are presented as mean ± SD, median, and interquartile range. Statistical differences were determined using the Mann–Whitney *U* test for two groups and the Steel–Dwass test for three or more groups. A *p* value < 0.05 was defined as statistically significant. All statistical analyses were performed using EZR (Saitama Medical Center, Jichi Medical University, Saitama, Japan), a graphical user interface for R (R Foundation for Statistical Computing, Vienna, Austria).

## 3 Results

### 3.1 Comparison of Aortic Calcium Deposition With Different Degrees of Hypoperfusion

Aortic wall hypoperfusion was induced in model animals by ligating the aorta over a catheter inserted into the lumen. Figure 1A shows the degree of hypoperfusion‐induced stress differed in each region of the aorta. To evaluate the relationship between the degree of hypoperfusion and calcium deposition, regional calcium deposition was assessed using Von Kossa staining. The area of calcium deposition in the media was significantly higher in Region 3 than that in the control region, Region 1 (Figure 1B–E,J). Calcium deposition was observed in some arteries in Regions 2 and 4, however, the averaged calcium deposition areas in Regions 2 and 4 were not significantly different from that in Region 1. The calcium deposition area in Region 4 tended to be higher than that in Region 2. The calcium deposition area was rarely observed in the adventitia, and there was no significant difference among the four regions (Figure 1B–E,K). The thickness of the arterial media in Region 3 was significantly lower than that in Region 1 (Figure 1F–I,L). Regions 2 and 4 showed no significant differences compared to Region 1.

### 3.2 Elemental Analysis in Von Kossa Staining‐Positive Areas (SEM/EDS)

**FIGURE 2:**
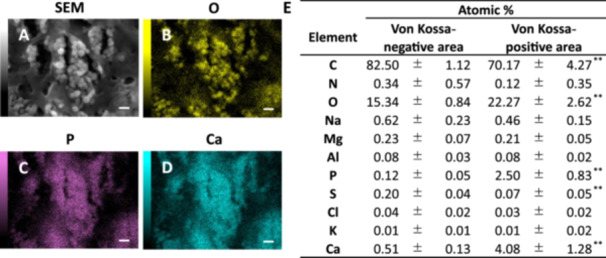
Elemental analysis of calcium deposits (SEM/EDS). (A) Representative SEM image of the Von Kossa‐positive area (scale bar = 1 μm). (B–D) Representative images of EDS analysis elemental mapping of the Von Kossa‐positive area (scale bar = 1 μm). (E) Comparison of atomic % of each element in EDS analysis. Values are shown as mean ± SD (*n* = 3). **p < 0.01, statistically significant.

Elemental analysis was performed using SEM/EDS to determine the elemental composition of the material deposited in the Von Kossa staining‐positive areas. Elements were measured in the Von Kossa staining‐negative area (Von Kossa −) and the Von Kossa staining‐positive area (Von Kossa +) of the aortic media, and the atomic percentages were compared. In the Von Kossa + area, a microcrystalline structure was observed, and the distribution of oxygen (O), phosphorus (P), and calcium (Ca) matched with the microstructure (Figure 2A–D). The atomic percentages of O, P, and Ca were significantly increased in the Von Kossa + area compared to those in the Von Kossa – area (Figure 2E). These results suggested that the material deposited in the Von Kossa + area was calcium phosphate crystals. Carbon (C) and sulfur (S) were significantly decreased in the Von Kossa + area compared to those in the Von Kossa – area.

### 3.3 Time‐Dependent Pathological Analysis in Region 3

**FIGURE 3:**
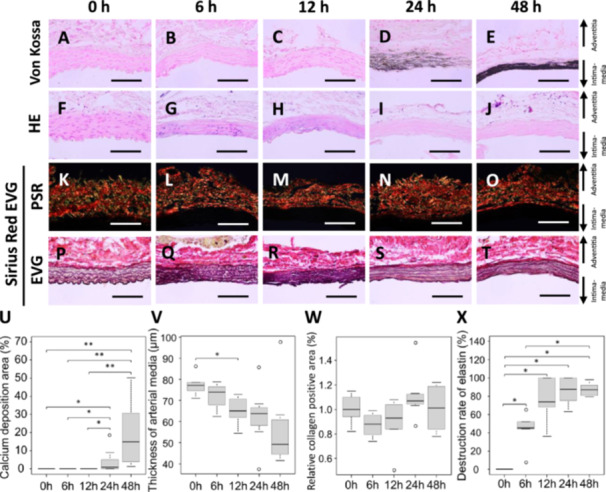
Time dependent of calcium phosphate deposition following induction of arterial wall hypoperfusion in Region 3. (A–E) Representative images of Von Kossa staining (scale bar = 100 μm). (F–J) Representative images of HE staining (scale bar = 100 μm). (K–O) Representative images of PSR staining (scale bar = 100 μm). (P–T) Representative images of EVG staining (scale bar = 100 μm). (U) Calcium deposition area in the media, (V) thickness of arterial media, (W) relative collagen positive area, and (X) destruction rate of elastin. (U, V) 0 h group (*n* = 6), 6 h group (*n* = 8), 12 h group (*n* = 8), 24 h group (*n* = 7), and 48 h group (*n* = 7). (W) 0 h group (*n* = 6), 6 h group (*n* = 8), 12 h group (*n* = 6), 24 h group (*n* = 7), and 48 h group (*n* = 6). (X) 0 h group (*n* = 6), 6 h group (*n* = 5), 12 h group (*n* = 6), 24 h group (*n* = 7), and 48 h group (*n* = 6). Values are shown as mean ± SD, median, and interquartile range. *p < 0.05, **p < 0.01, statistically significant.

Because the Von Kossa staining‐positive area was significantly higher in Region 3 than that in other regions, subsequent pathological analyses were focused on Region 3. The Von Kossa staining‐positive area in the media significantly increased at 24 and 48 h compared to 0 h (Figure 3A–E,U). The thickness of the arterial media tended to decrease over time (Figure 3F–J,V). There was no significant difference in the collagen fiber‐positive area among the groups (Figure 3K–O,W). On the contrary, the elastin destruction rate significantly increased at 6, 12, 24, and 48 h compared to 0 h (Figure 3P–T,X).

### 3.4 The Formation of Calcium Phosphate Crystal in the Microvoids in the Arterial Media

**FIGURE 4:**
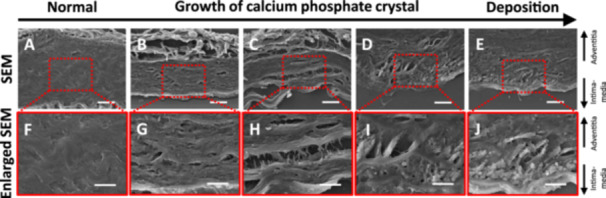
SEM images of the arterial media following induction of arterial wall hypoperfusion. Calcium phosphate deposition process following induction of arterial wall hypoperfusion. (A–E) Representative SEM images (scale bar = 20 µm). (F–J) Enlarged images of SEM images (scale bar = 10 µm).

Time‐dependent observations suggested a progressive pathological process in which medial thinning occurred first following hypoperfusion, subsequently leading to calcium phosphate deposition. To investigate the microstructural changes associated with the process, the arterial media were observed using SEM. Before the induction of arterial wall hypoperfusion, the media layer displayed a dense structure composed of tightly packed fibers and cells (Figure 4A,F). After induction of hypoperfusion, the fibers within the media layer became disorganized and loose, and the formation of microvoids was observed (Figure 4B,G). These microvoids progressively enlarged (Figure 4C,H), providing scaffolds for the deposition of calcium phosphate crystals (Figure 4D,I). Eventually, the microvoids became filled with calcium phosphate deposits (Figure 4E,J).

### 3.5 Time‐Dependent Observation of the Nuclear‐Positive Area in the Arterial Media Layer

**FIGURE 5:**
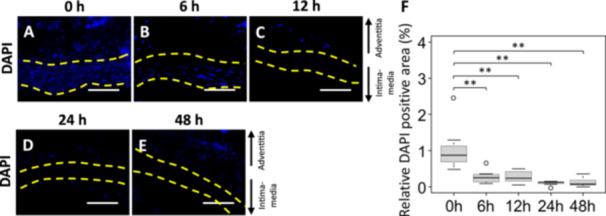
Time dependent of nuclear positive areas following induction of arterial wall hypoperfusion in Region 3. (A–E) Representative images of DAPI staining. The yellow dotted line indicates the extent of the arterial media (scale bar = 100 µm). (F) Quantification of the DAPI positive area in the arterial media. 0 h group (*n* = 8), 6 h group (*n* = 8), 12 h group (*n* = 7), 24 h group (*n* = 7), and 48 h group (*n* = 7). Values are shown as mean ± SD, median, and interquartile range. **p < 0.01, statistically significant.

The thinning of the media and the formation of microvoids after hypoperfusion suggested pathological changes in the cells that compose the media, so the nuclei in the media layer were stained with DAPI. The DAPI‐positive area was significantly decreased at 6, 12, 24, and 48 h compared to 0 h (Figure 5).

### 3.6 Changes in Osteoblast‐Like Cell Markers and Osteoclast Markers in VSMCs

**FIGURE 6:**
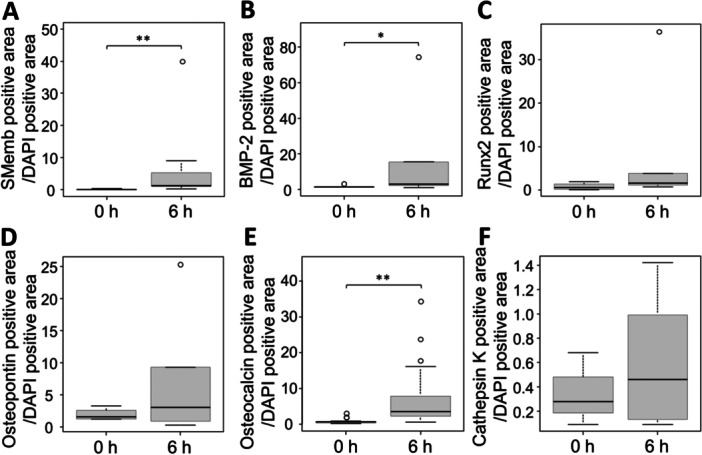
Time‐dependent changes in osteoblast‐like and osteoclast markers in the arterial media of Region 3. Comparison of the areas in the arterial media that were positive for fluorescent immunostaining of (A) SMemb, (B) BMP‐2, (C) Runx2, (D) Osteopontin, (E) Osteocalcin, and (F) Cathepsin K at different time points. To compare living cells, the positive areas of each marker were normalized to the DAPI‐positive areas. Statistical analyses were performed using values obtained from three or more regions per sample. (A) 0 h group (*n* = 5), 6 h group (*n* = 7), (B) 0 h group (*n* = 5), 6 h group (*n* = 8), (C, D) 0 h group (*n* = 4), 6 h group (*n* = 6), (E) 0 h group (*n* = 5), 6 h group (*n* = 5), (F) 0 h group (*n* = 3), 6 h group (*n* = 7). Values are shown as mean ± SD, median, and interquartile range. *p < 0.05, **p < 0.01, statistically significant.

Changes in factors involved in calcification were evaluated by immunostaining. Because the results of DAPI staining showed that pathological changes occurred in cells 6 h after induction of arterial wall hypoperfusion, this analysis was performed at 6 h. To compare living cells, the positive areas of each marker were normalized to the DAPI‐positive area. In the arterial media layer, the positive areas of SMemb and BMP‐2, which are osteoblast‐like cell markers in VSMCs, were significantly increased at 6 h compared to 0 h (Figure 6A,B, Figure S1). The positive areas of Runx2 and Osteopontin, which are downstream factors of BMP‐2, tended to increase at 6 h compared to 0 h (Figure 6C,D, Figures S2A–F, S3). The positive area of Osteopontin was significantly increased at 12 h compared to 0 h (Figure S3). The positive areas of Osteocalcin, a downstream factor of Runx2, significantly increased at 6 h compared with 0 h (Figure 6E, Figure S2G–L). Colocalization was observed between SMemb and BMP‐2, and between SMemb and osteocalcin, respectively (Figure S4). The area positive for Cathepsin K, a marker for osteoclasts, was not significantly different at 6 h compared to 0 h (Figure 6F, Figure S2M–R).

## 4 Discussion

Our study showed that aortic wall hypoperfusion can be the initiation factor of ectopic calcium phosphate deposition in the arterial media, which is an early lesion of medial calcification and sclerosis.

In this study, the artery where hypoperfusion was induced in the aortic wall was divided into four regions to investigate the relationship between the degree of induced condition of aortic hypoperfusion and calcium phosphate deposition. Region 1 was the control region that was hardly affected with induction of hypoperfusion by intercalators: catheter and ligature. Based on the positions of the catheter, ligature, and the aorta, Region 3 was the most severely affected by the induction of hypoperfusion. Regions 2 and 4 were relatively less affected by the induction of hypoperfusion compared to Region 3. The results of this study, which showed association between the degree of hypoperfusion and calcium phosphate deposition, suggest that hypoperfusion‐resulting events, namely hypoxia and/or malnutrition in the arterial wall can be associated with calcium phosphate deposition in the aorta. It has been reported that the progression of arterial medial calcification is associated with hypoxia, and calcium deposition on VSMCs or the aorta increases both in vitro and ex vivo under hypoxic conditions [18] and that hypoxia‐inducible factor‐1α increased with the induction of hypoperfusion in the arterial wall [12, 13]. These results suggest that hypoxia plays an important role in the deposition of calcium phosphate in the hypoperfusion‐induced aorta. Under the conditions of this study, the Von Kossa‐positive area in Region 4 tended to be higher than that in Regions 2 (48 h after the induction of hypoperfusion). At 24 h, the Von Kossa‐positive area in Region 4 was significantly higher than that in Region 1, while there was no significant difference between Region 2 and Region 1 (data not shown). The difference between Regions 2 and 4 might be attributed to the supply of oxygen; the oxygen supplied to Region 4 from the lumen is via blood backflow, so it may be that Region 4 is more prone to hypoxia than Region 2. In humans, oxygen is supplied to the arterial media through both the vascular lumen and the vasa vasorum [19]. Luminal thrombus is a factor that causes hypoxia in the arterial inner wall [20], and chronic thrombus has been reported to be associated with medial arterial calcification [21]. A vicious cycle might exist in which arterial dysfunction caused by calcium phosphate deposition in the media promotes thrombus formation.

The formation and progression of medial arterial calcification is accompanied by the transformation of VSMCs into osteoblast‐like cells which release matrix vesicles containing phosphate and calcium ions [22]. The reaction products of the ions form crystal nuclei on the denatured elastic fibers, and the crystals grow to form calcified areas mainly composed of hydroxyapatite [23, 24]. In this study, we observed flattening of elastic fibers and death of medial cells prior to calcium phosphate deposition, followed by the formation of microvoids in the media. The resulting microvoids were then used as scaffolds for calcium phosphate crystal formation. We previously reported the increased amounts and activities of matrix metalloproteinase (MMP)‐2 and MMP‐9 in the arterial wall after induction of hypoperfusion [13, 25]. These data suggest that hypoperfusion‐induced increased activities of MMPs and death of VSMCs induced the formation of medial microvoid as a spatial origin of calcium phosphate crystal formation. At 6 h after the induction of hypoperfusion, degeneration of elastic fibers, a reduction in the nuclear‐positive area within the media, and the expression of osteoblast‐like cell markers SMemb and BMP‐2 were observed. In addition, Osteopontin, a downstream factor of BMP‐2 [26], was observed 12 h after hypoperfusion, and calcium phosphate deposition was observed 24 h after induction. These results of time‐dependent pathological analysis suggest that the change in the aortic wall environment under hypoperfusion simultaneously induces cell death and transformation of VSMCs, which ultimately leads to calcium phosphate deposition. We observed advanced pathological changes including medial thinning, destruction of elastic fibers, loss of nuclei, circumferential Von Kossa‐positive areas, and calcium phosphate crystal growth in the arterial wall 8 weeks after induction of hypoperfusion (Figure S5). This result indicates that cell‐dependent processes are involved in the early formation of calcium phosphate crystals, and then cell‐independent processes become dominant, which can induce the long‐term growth of calcium phosphate crystals. Because Region 3 is in an environment where the circulation originating from the lumen and vasa vasorum is completely cut off, the results of this study indicate that crystals of calcium phosphate can be generated solely from substances in the intracellular or pericellular environment, without being influenced by blood components. Since local hypoperfusion can occur throughout the body other than the aortic wall, the process of localized hypoperfusion might explain part of the mechanism of ectopic calcification formation frequently observed in humans.

In conclusion, this study demonstrated the causal relationship between arterial wall hypoperfusion and calcium phosphate deposition. Animal model used in this study allows for the time‐dependent observation of pathologies of the aortic wall with different degrees of hypoperfusion, which can contribute to further understanding of the mechanism of medial arterial calcification formation. Our experimental animal model suggests that hypoperfusion leads to decrease and phenotypic alteration of VSMCs, and degeneration of elastin fibers. The resulting microvoids provide scaffolds for calcium phosphate crystal formation, eventually growing into areas of calcification. A limitation of this study is that the relationship between blood components and calcium phosphate deposition was not discussed because an isolated space, Region 3, was used as the research model. In future studies, the influence of blood components should be evaluated by analyzing Regions 2 or 4. The results of this study do not exclude the involvement of blood components in calcium phosphate deposition. Another limitation of this study is that observation of this study was focused on Region 3 where severe aortic ischemia was mimicked, a condition that typically requires several years to develop in humans. Although our data show that calcium phosphate crystals can rapidly form after the induction of severe hypoxic conditions, the growth process under mild hypoxia still needs to be investigated. Further research is necessary to elucidate the complex relationship among blood components, aortic wall hypoperfusion, and calcium phosphate deposition.

## Author Contributions

**Tomoko Sumi:** performed animal experiments, analyzed the data, wrote the manuscript, and secured the funding. **Mayo Higashihara, Takuma Takeda, Taichi Imai, and Yuna Tamura:** performed animal experiments and edited manuscripts. **Tatsuya Moriyama:** edited the manuscript. **Nobuhiro Zaima:** designed the studies, wrote and edited the manuscript, and secured the funding.

## Conflicts of Interest

The authors declare no conflicts of interest.

## Supporting information

251114 MAC animal model_Supplementary figure.

supmat.

## Data Availability

All data are contained within the main article and Supplementary Information.
