# Supplementary figures and images for: Pathological Evidence From an Experimental Rat Model Demonstrates That Aortic Hypoperfusion Contributes to the Development of Medial Arterial Calcification
Source: Pathol Int. 2025 Dec 30;76(1):e70077. doi: 10.1111/pin.70077 (PMC12835961; doi:10.1111/pin.70077)

**Fig. S1**

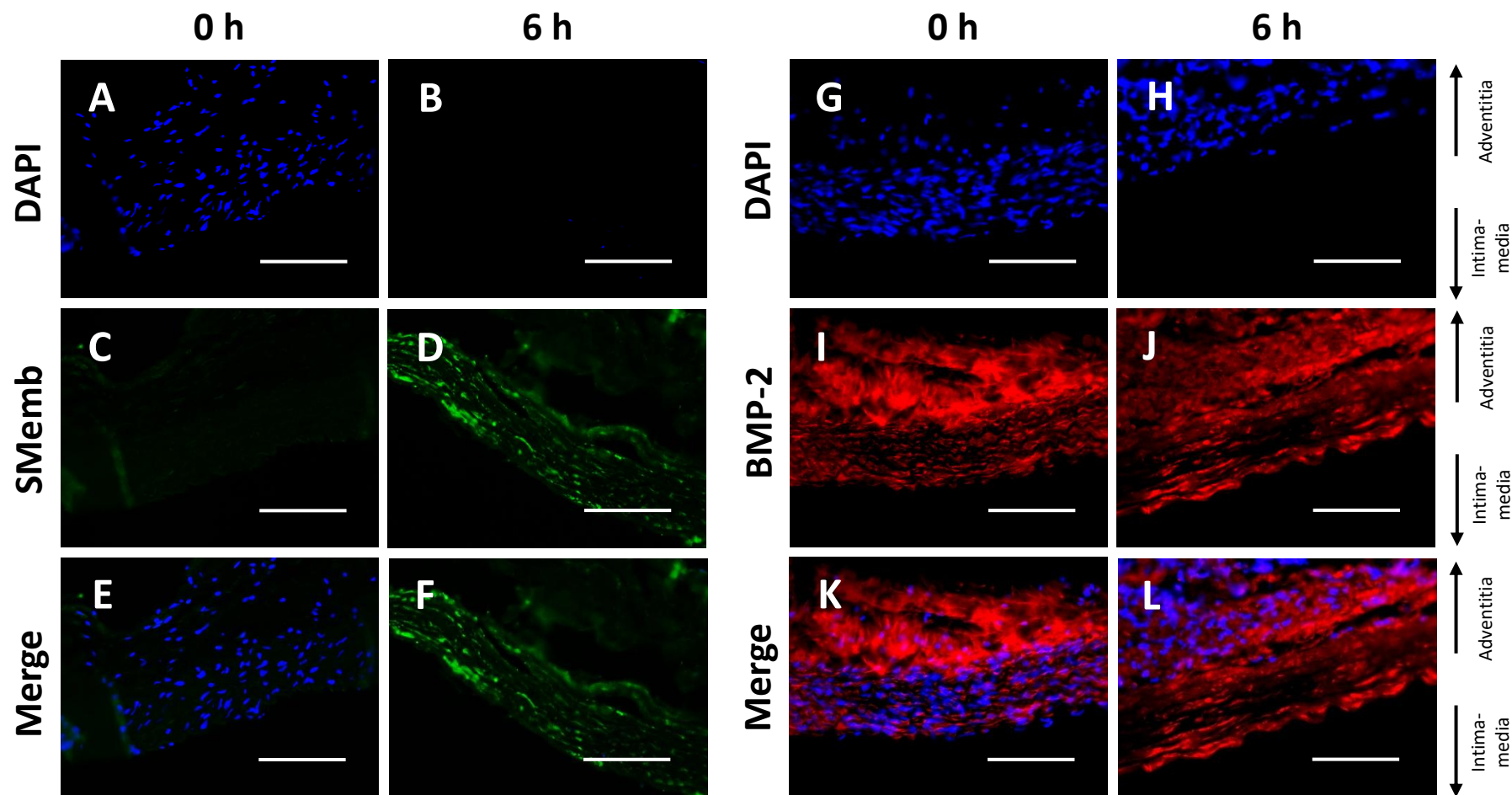

**Fig. S2**

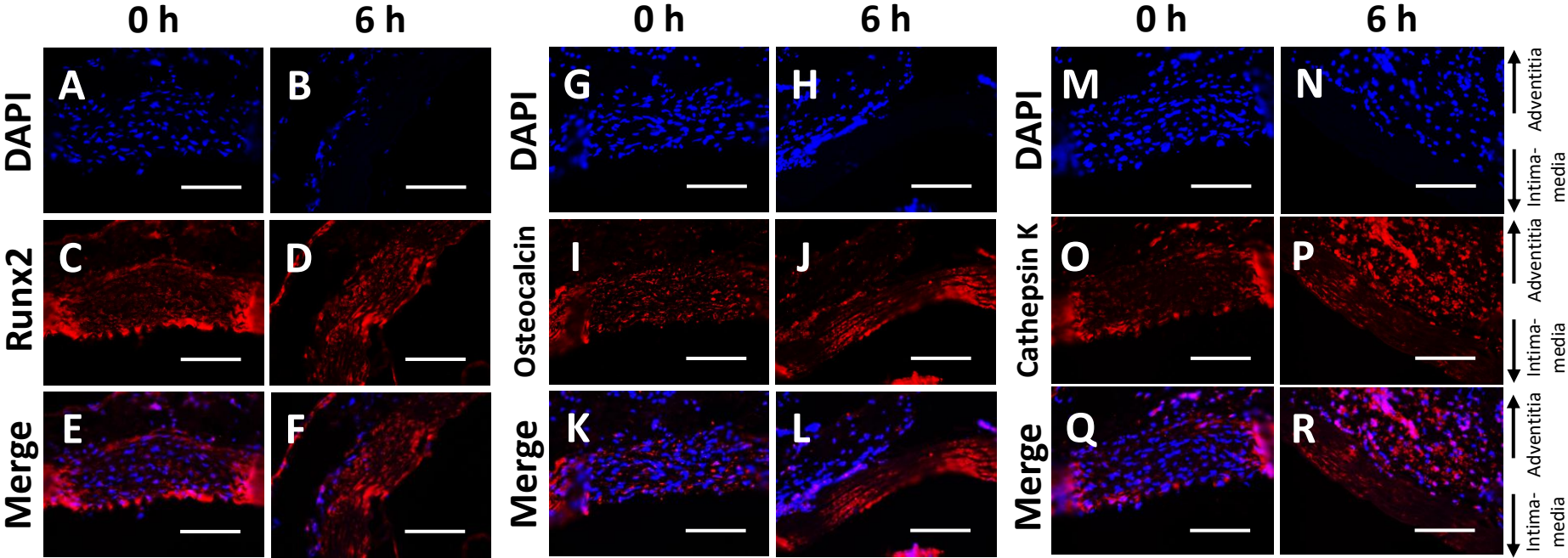

**Fig. S3**

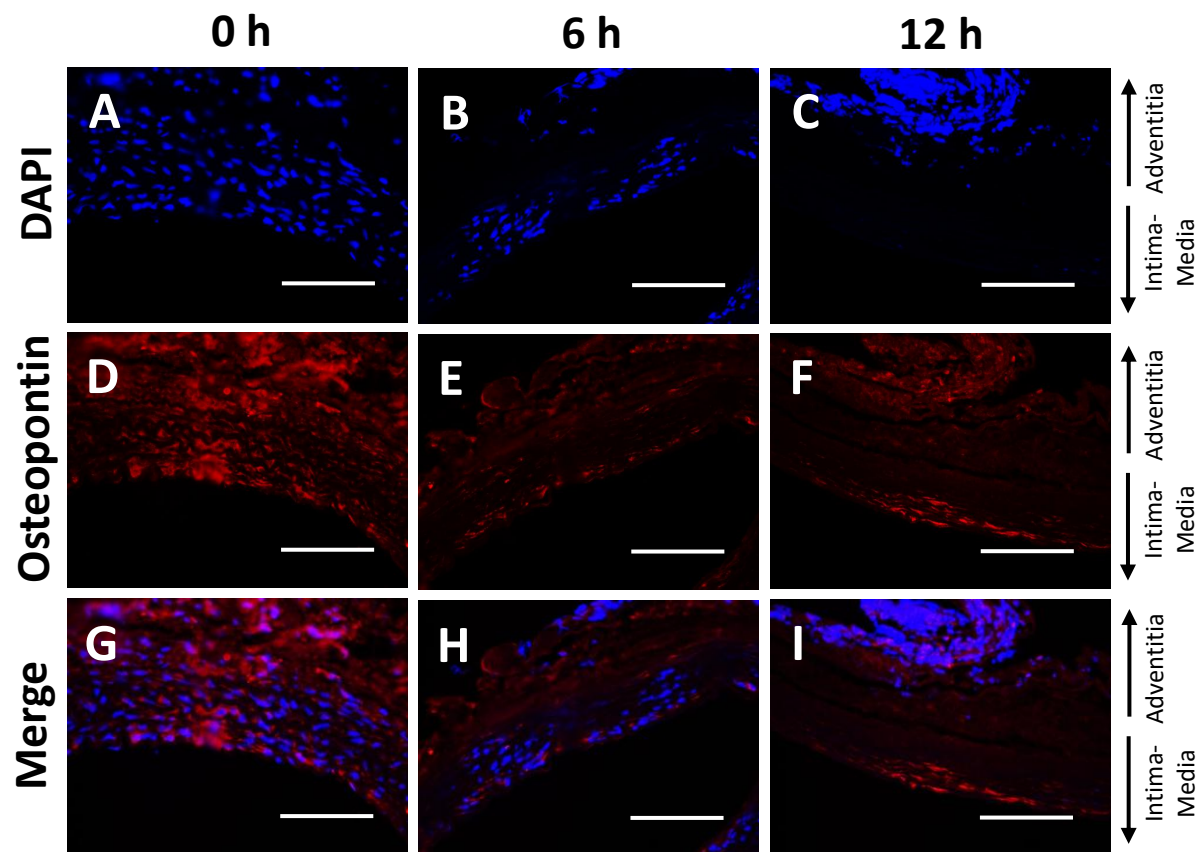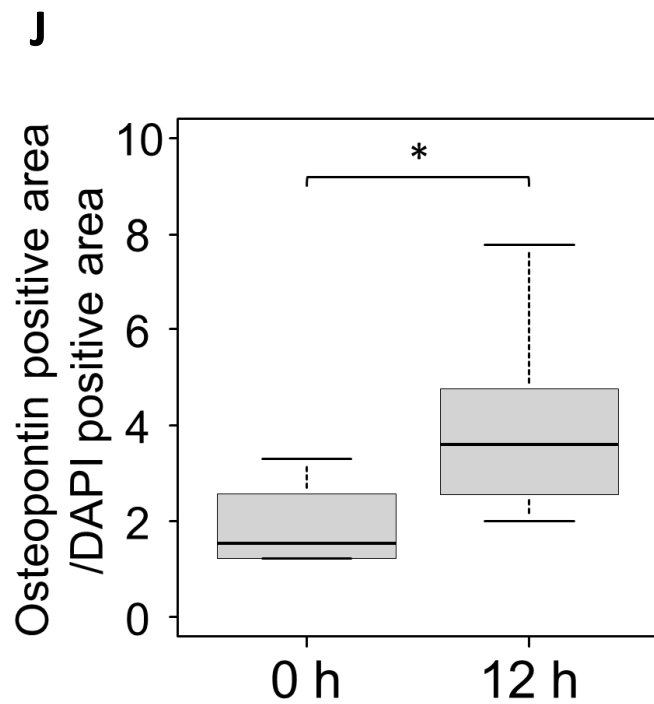

**Fig. S4**

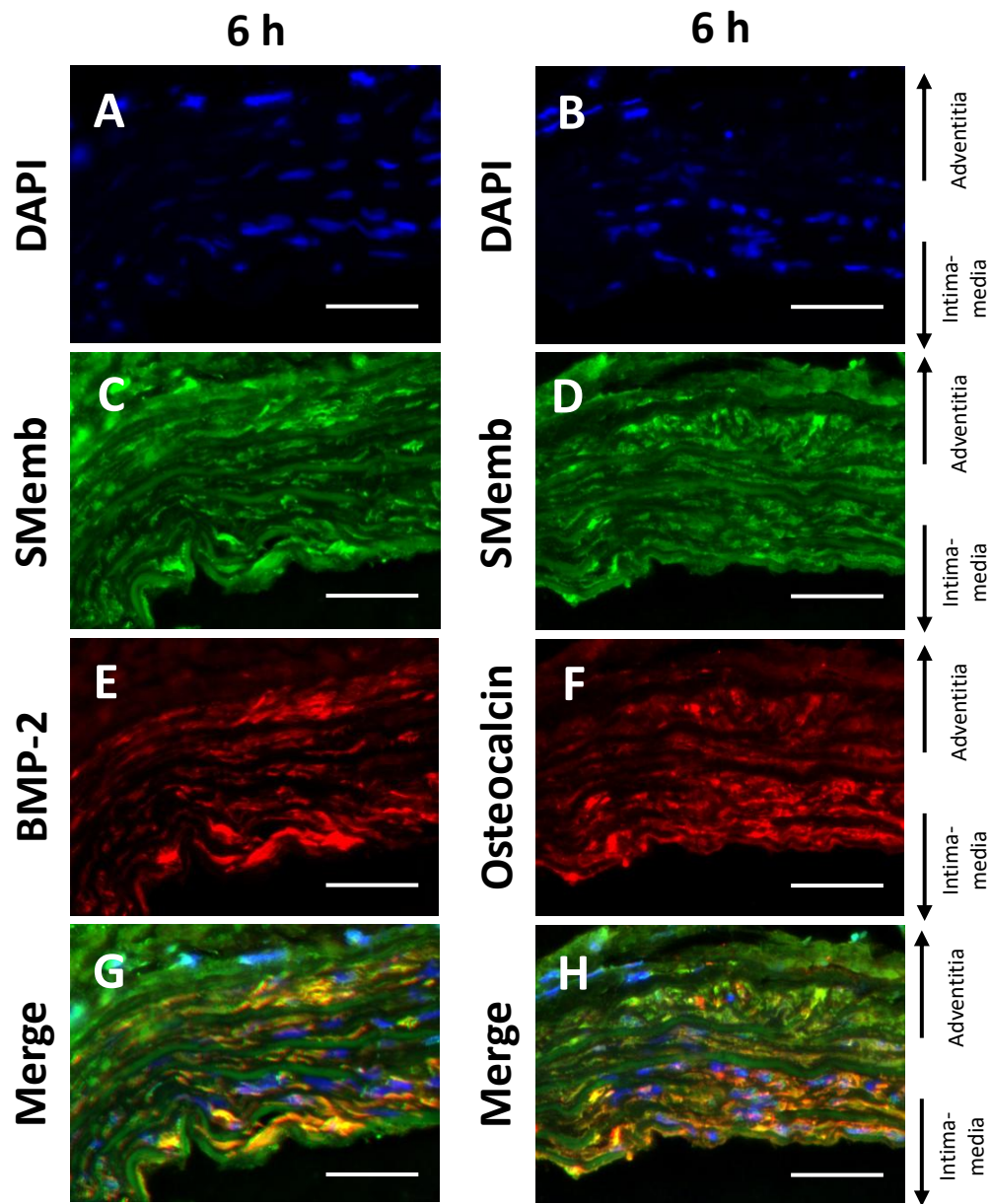

**Fig. S5**

**Von Kossa**

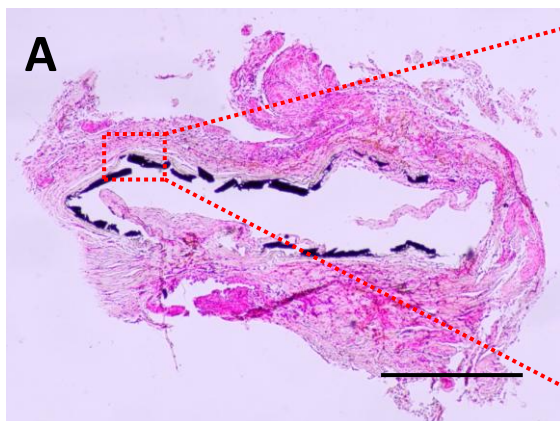

**SEM**

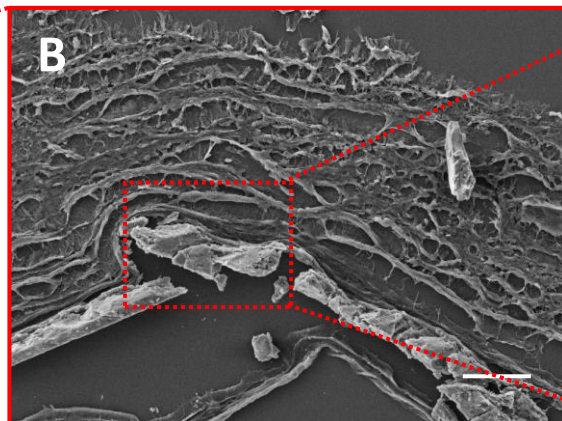

**Enlarged SEM**

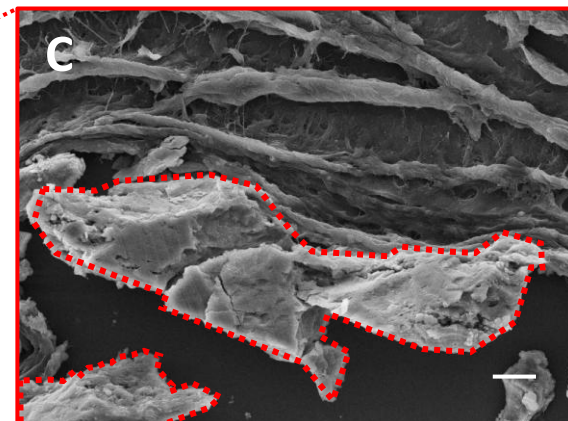

**O**

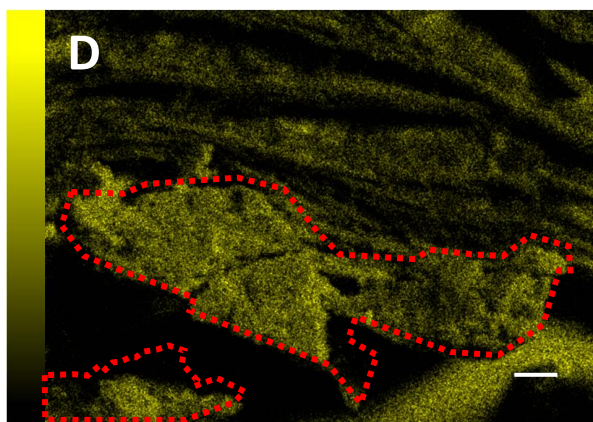

**P**

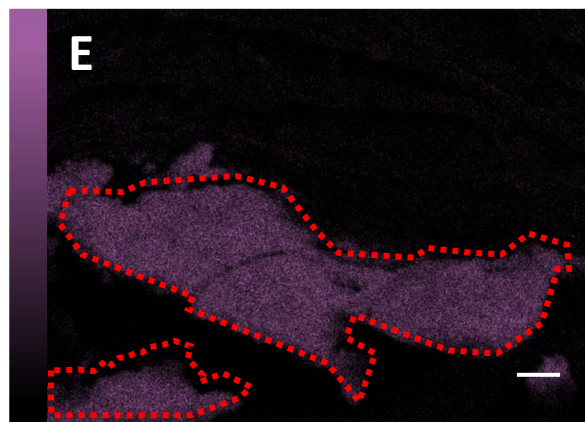

**Ca**

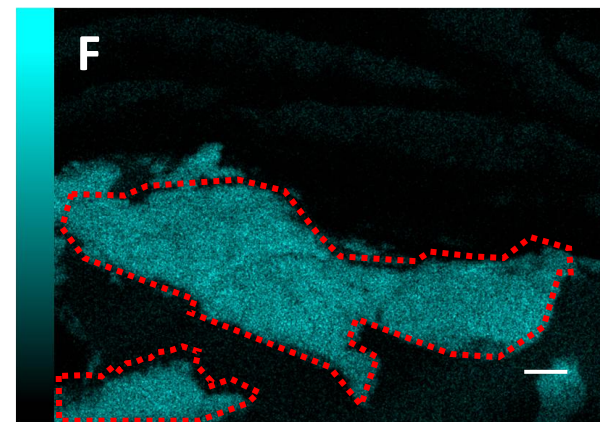

Supplement: Supplementary file 1 — 251114 MAC animal model_Supplementary figure. [file PIN-76-0-s001.pdf]
