# Supplementary material for: Pathological Evidence From an Experimental Rat Model Demonstrates That Aortic Hypoperfusion Contributes to the Development of Medial Arterial Calcification
Source: Pathol Int. 2025 Dec 30;76(1):e70077. doi: 10.1111/pin.70077 (PMC12835961; doi:10.1111/pin.70077)
Supplement: Supplementary file 2 — supmat. [file PIN-76-0-s002.docx]

**Fig. S1.**

**Staining images of osteoblast-like cell markers (SMemb, BMP-2) in the arterial media.**

SMemb immunostaining: (A, B) Representative DAPI staining images, (C, D) Representative SMemb staining images, (E, F) Merge images of DAPI and SMemb (scale bar = 100▒µm). BMP-2 immunostaining: (G, H) Representative DAPI staining images. (I, J) Representative BMP-2 staining images. (K, L) Merge images of DAPI and BMP-2 (scale bar = 100▒µm).

**Fig. S2.**

**Staining images of osteoblast-like cell marker (Runx2), Osteocalcin and osteoclast marker (Cathepsin K) in the arterial media.**

Runx2 immunostaining: (A, B) Representative DAPI staining images, (C, D) Representative Runx2 staining images, (E, F) Merge images of DAPI and Runx2 (scale bar = 100▒µm). Osteocalcin immunostaining: (G, H) Representative DAPI staining images, (I, J) Representative Osteocalcin staining images, (K, L) Merge images of DAPI and Osteocalcin (scale bar = 100▒µm). Cathepsin K immunostaining: (M, N) Representative DAPI staining images. (O, P) Representative Cathepsin K staining images. (Q, R) Merge images of DAPI and Cathepsin K (scale bar = 100▒µm).

**Fig. S3.**

**Time depends on osteoblast-like cell marker (Osteopontin) in the arterial media layer.**

(A–C) Representative images of DAPI staining, (D–F) Representative images of Osteopontin, (G–I) Merge images of DAPI and Osteopontin (scale bar = 100▒µm). (J) Comparison of the areas in the arterial media that were positive for fluorescent immunostaining of Osteopontin in the arterial media. To compare living cells, the positive areas of each marker were normalized to the DAPI-positive areas. Statistical analyses were performed using values obtained from three or more regions per sample. 0▒h group (n▒=▒4), 12▒h group (n▒=▒6). Values are shown as mean▒±▒SD, median, and interquartile range (n▒=▒5). **p*▒<▒0.05, statistically significant.

**Fig. S4.**

**Staining images of SMemb, BMP-2 and Osteocalcin in the arterial media.**

(A, B) Representative DAPI staining images, (C, D) Representative SMemb staining images, (E) Representative BMP-2 staining images, (F) Representative Osteocalcin staining images, (G, H) Merge images (scale bar = 50▒µm).

**Fig. S5.**

**Calcium deposition 8 weeks after induction of arterial wall hypoperfusion (SEM/EDS).**

(A) Representative images of Von Kossa staining (scale bar = 100▒µm). (B) Representative images of SEM (scale bar = 50▒µm). (C) Enlarged images of SEM images (scale bar = 100▒µm). (D–F) Representative images of EDS analysis (scale bar = 10▒µm).
